# Bivariate analysis of basal serum anti-Müllerian hormone measurements and human blastocyst development after IVF

**DOI:** 10.1186/1477-7827-9-153

**Published:** 2011-12-02

**Authors:** E Scott Sills, Gary S Collins, Adam C Brady, David J Walsh, Kevin D Marron, Alison C Peck, Anthony PH Walsh, Rifaat D Salem

**Affiliations:** 1 Division of Reproductive Endocrinology, Pacific Reproductive Center; Irvine, California, USA; 2 Centre for Statistics in Medicine, Wolfson College Annexe, University of Oxford; Oxford, UK; 3 Department of Medicine, University of Massachusetts School of Medicine; Worcester, Massachusetts, USA; 4 Division of Reproductive Endocrinology, The Sims Institute/Department of Obstetrics & Gynaecology, School of Medicine, Royal College of Surgeons in Ireland; Dublin, Ireland

**Keywords:** serum AMH, IVF, embryo development, blastocyst transfer

## Abstract

**Background:**

To report on relationships among baseline serum anti-Müllerian hormone (AMH) measurements, blastocyst development and other selected embryology parameters observed in non-donor oocyte IVF cycles.

**Methods:**

Pre-treatment AMH was measured in patients undergoing IVF (*n *= 79) and retrospectively correlated to *in vitro *embryo development noted during culture.

**Results:**

Mean (+/- SD) age for study patients in this study group was 36.3 ± 4.0 (range = 28-45) yrs, and mean (+/- SD) terminal serum estradiol during IVF was 5929 +/- 4056 pmol/l. A moderate positive correlation (0.49; 95% CI 0.31 to 0.65) was noted between basal serum AMH and number of MII oocytes retrieved. Similarly, a moderate positive correlation (0.44) was observed between serum AMH and number of early cleavage-stage embryos (95% CI 0.24 to 0.61), suggesting a relationship between serum AMH and embryo development in IVF. Of note, serum AMH levels at baseline were significantly different for patients who did and did not undergo blastocyst transfer (15.6 vs. 10.9 pmol/l; *p *= 0.029).

**Conclusions:**

While serum AMH has found increasing application as a predictor of ovarian reserve for patients prior to IVF, its roles to estimate *in vitro *embryo morphology and potential to advance to blastocyst stage have not been extensively investigated. These data suggest that baseline serum AMH determinations can help forecast blastocyst developmental during IVF. Serum AMH measured before treatment may assist patients, clinicians and embryologists as scheduling of embryo transfer is outlined. Additional studies are needed to confirm these correlations and to better define the role of baseline serum AMH level in the prediction of blastocyst formation.

## Background

Anti-Müllerian hormone (AMH), a dimeric glycoprotein member of the transforming growth factor-*β *superfamily, is a product of the granulosa compartment involved in regulation of early ovarian follicular growth and cyclic follicular selection [1,2]. AMH has a highly conserved promoter region and plays a crucial role in mammalian reproduction [3]. Recent data have identified serum AMH as a useful marker for ovarian reserve, as it correlates with number of primordial follicles and declines with advanced reproductive age [4]. AMH is favoured as an ovarian reserve test because of its relative constancy throughout the menstrual cycle [5,6], and its tendency to be unchanged despite GnRH-agonist pituitary down-regulation [7] or pregnancy [8]. These aspects of AMH have encouraged its use before treatment to elucidate diminished reserve associated with ovarian ageing [9,10]. However, a less well-studied application for pre-IVF serum AMH is its ability to forecast the developmental potential of embryos derived from IVF. If pre-treatment AMH levels could assist in the choice between d3 vs. d5 transfer, this would better manage patient expectations and help establish provisional embryo transfer schedules. By correlating embryo morphology noted during *in vitro *culture with serum AMH levels measured before planned blastocyst transfer in IVF patients, the present investigation sought to clarify this question.

## Methods

### Study patients

Between September 2009 - December 2009, serum AMH levels were measured in patients (*n *= 79) commencing non-donor gamete IVF at a single centre. All serum samples were collected during the early follicular phase (d≤7) within three months of controlled ovarian hyperstimulation for IVF. Treatment cycles incorporating frozen embryo transfer were excluded. All patients were in good general health and had normal pre-treatment laboratory testing; each patient was assessed by a consultant physician before IVF and written informed consent was obtained prior to study enrolment. Normal sperm concentration, motility, and morphology was observed for each male partner in this study group, and sperm DNA fragmentation analysis confirmed sperm chromatin fragmentation was < 25%. While this sample included no severe male factor cases, a limited number of couples (*n *= 3) requested "upfront ICSI" on an individual basis. All patients in this study were non-smokers. For females, baseline (pre-treatment) serum AMH values were correlated with the following intercycle parameters: Female patient age (yrs), terminal serum E_2_, total number of oocytes retrieved, number of metaphase II (MII) oocytes retrieved, number of early cleavage stage embryos produced after fertilisation, number of embryos with ≥7 cells present on d3, and the number of blastocysts that had developed by d5. Neither basal antral follicle count, serum d3 FSH, nor ampoules/type of gonadotropins consumed during follicular recruitment were recorded for analysis, although clinical pregnancy rate was also noted as a secondary endpoint. Because this was a retrospective study, institutional review board approval was not required. For all patients, pituitary downregulation was achieved with twice-daily 200 mcg intranasal nafarelin acetate (Synarel^®^; Phamacia Ltd., Milton Keynes, UK) followed by gonadotropin administration and office monitoring as described previously [11].

### AMH assay procedure

Each patient provided one specimen by peripheral venipuncture which was assayed for AMH in duplicate via MIS/AMH ELISA DSL-10-14400 (Diagnostic Systems Laboratories, Inc.; Webster, Texas USA). This device used an enzymatically amplified two-site immunoassay where standards, controls, and samples were incubated in microtitration wells coated with anti-AMH antibody. After incubation and washing, anti-AMH detection antibody labeled with biotin was added to each well. Streptavidin horseradish peroxidase (HRP) was added to wells following a secondary incubation/washing. Tetramethylbenzidine (TMB) was introduced after a third incubation and washing procedure, followed by addition of an acidic stopping solution. Enzymatic turnover of substrate was determined by dual wavelength absorbance measurement at 450 nm and 600-630 nm (measured absorbance is directly proportional to sample AMH content). Standards were processed concurrently with experimental samples to determine serum AMH for each specimen. The minimum detectable level of AMH in serum was 0.04 pmol/ml, with intra- and interassay coefficients of variation of < 5% and < 8%, respectively.

### Assessment of embryo development

Immediately after retrieval oocyte-cumulus complexes were placed into Universal IVF medium (MediCult; Jyllinge, Denmark), with insemination (including ICSI) also carried out using this reagent under washed liquid paraffin oil (MediCult, Denmark). Culture was maintained to d5 in microdrops of BlastAssist media I and II (MediCult, Denmark) under washed paraffin oil in a 5%CO2 + 5%O2 atmosphere at 95% humidity. Embryo development was observed via multi-port inverted optical microscope fitted with Hoffman contrast modulation (Nikon TE300; Tokyo, Japan). Daily assessments of embryos were recorded at 200-400× magnification; d5 blastocysts selected for *in utero *transfer featured a well-defined inner cell mass and highly cellular, expanding trophoectoderm [12].

### Statistical analysis

The Mann-Whitney test was used to test differences, as the outcomes were skewed (non-normal distribution). Differences measured at *p *< 0.05 were considered significant; differences between groups were reported as pseudomedians (*i.e*., median of differences between samples).

## Results

**Figure 1:**
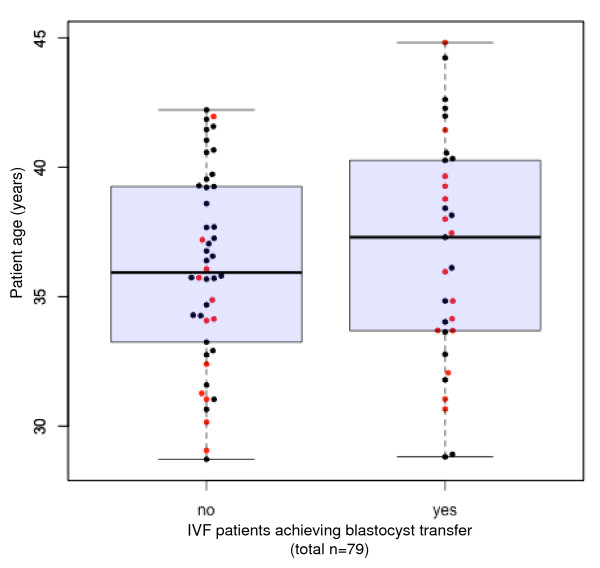
**Basal AMH and female age**. Distribution of serum anti-Mullerian hormone (AMH) levels as a function of female age among 79 IVF patients (pregnancies shown in red).

**Figure 2:**
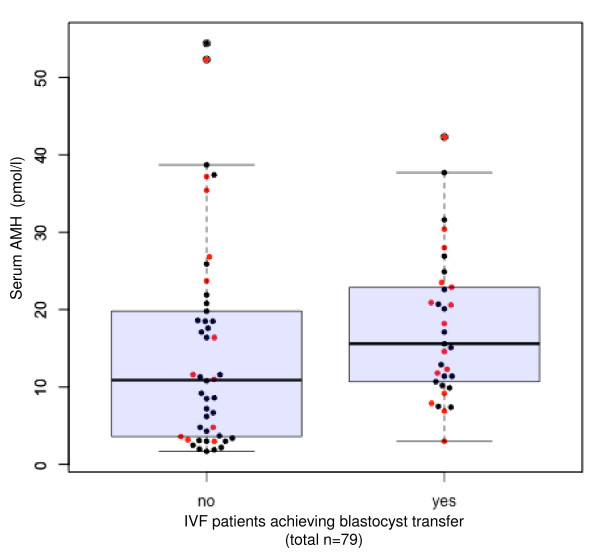
**Basal AMH and blastocyst development**. Relation between serum anti-Mullerian hormone (AMH) levels at baseline and subsequent potential to undergo blastocyst transfer (+BT; *n *= 33; no BT = 46 patients). *Note*: Pregnancies are shown in red.

In this study group, median (IQR 25;75) female patient age was 36.1 (33.7; 39.4) overall; patient ages among those who did (*n *= 27) and did not (*n *= 52) attain clinical pregnancy after embryo transfer were 34.8 (32.2; 37.7) and 37.1 (34.2; 40.3), respectively (*p *= 0.073). No analysed IVF cycles were complicated by ovarian hyperstimulation syndrome and there were no cycle cancellations during the study interval. As summarised in Table 1, pre-treatment serum AMH was significantly higher among patients attaining blastocyst transfer compared to those who did not (15.6 [10.7;22.9] vs. 10.9 [3.6;19.5]; *p *= 0.029). Consistent with previous reports [4,5], our data confirmed the inverse relationship between age and basal serum AMH (correlation -0.48; 95% CI -0.63 to -0.29). Figure 1 depicts the relationship between patient age and capacity for blastocyst transfer. As shown in Figure 2, median pre-treatment serum AMH levels were significantly different for patients who did undergo blastocyst transfer and those who did not (15.6 vs. 10.9 pmol/l; *p *= 0.029 by Mann-Whitney test). While median patient age was higher among patients who underwent blastocyst transfer compared to patients who did not, this difference was not significant (37.3 vs. 35.9; *p *= 0.509). Moreover, basal serum AMH levels were generally higher among patients who attained a clinical pregnancy, but this difference was not significant (16.4 vs. 11.4 pmol/l; *p *= 0.14 by Mann-Whitney test). In this study population, the observed clinical pregnancy rate was 27/79 per embryo transfer (34.2%).

**Table 1:** Blastocyst development *in vitro *compared with basal serum AMH and selected clinical/embryology parameters among IVF patients (*n *= 79)

	No blastocyst (*n *= 46)	Blastocyst (*n *= 33)	Difference (95% CI)	*p*^1^
Serum AMH (pmol/l)	10.9 (3.6, 19.5)	15.6 (10.7, 22.9)	4.9 (0.6 to 8.9)	0.029
Age (yrs)	35.9 (33.5, 39.2)	37.3 (33.7, 40.3)	0.7 (-1.4 to 2.7)	0.509
Terminal E_2 _(pmol/l)	4084 (2996, 5164)	7282 (3572, 10770)	2335 (-260 to 6361)	0.121
Oocytes retrieved (total *n*)	7 (4, 17)	10 (7, 13)	3 (1, 5)	0.004
MII oocytes retrieved (*n*)	5 (3, 8)	9 (6, 12)	3 (2, 5)	0.006
Early cleavage embryos (*n*)	3.5 (2, 6)	6 (5, 9)	3 (2, 4)	0.002
≥7-cell embryos on d3 (*n*)	1.5 (0, 3)	2 (1, 4)	1 (0, 2)	0.07

^1^by Mann-Whitney *U *test

*Notes: *All data presented as mean (IQR 25,75); AMH = anti-Müllerian hormone; E_2 _= estradiol;

MII = metaphase II; d3 = post-fertilisation day three

## Discussion

This investigation is the first to show a positive, albeit weak, correlation between baseline serum AMH levels and subsequent blastocyst development during IVF. Although serum AMH has found increasing application as a predictor of ovarian reserve, its role in forecasting *in vitro *embryo morphology and developmental potential to the blastocyst stage is still emerging. For example, Riggs *et al *[13] found AMH to be a useful biomarker to predict low vs. high responders among oocyte donors, but not predictive of embryo morphology or pregnancy outcome in the recipient population. Similarly, while an earlier study reported basal serum AMH as useful in predicting oocyte number and quality, further cleavage up to the blastocyst stage was not affected by AMH [14]. In contrast, the current investigation suggests that baseline serum AMH determinations can indeed be extended to estimate blastocyst developmental potential during IVF. A trend of somewhat lower patient age where no blastocyst transfer was possible represents an unexpected finding from our study, and underscores the limitations of using patient age alone as a method to predict blastocyst development.

Clinicians and researchers continue to search for improved methods to estimate ovarian reserve and predict reproductive outcome. Serum AMH may help solve this challenge, as it is maximal in females at puberty but progressively decays throughout reproductive life [15,16]. Indeed, physiologic menopause or surgical removal of functional ovaries will render serum AMH essentially undetectable within days [4,17]. More recently, specific polymorphisms of the AMH receptor have been implicated as additional modulators of sex-steroid activity [18]. Early determination of reserve is useful not only because this information helps guide patient counselling, but it also assists in calibrating the gonadotropin doses for IVF patients. Risks of poor follicular recruitment [19] and ovarian hyperstimulation [20,21] would be minimised by accurate and reliable prediction of ovarian response in IVF. Since serum AMH levels might help estimate ovarian response for non-IVF patients as well [22], this test has emerged as an increasingly important element of basic fertility assessments. When best to assess serum AMH has also been studied, but baseline measurement seems to be the most predictive marker for ovarian response [23]. Whether or not serum AMH can be validated as the definitive biophysiologic test to predict the age of menopause remains the subject of active study and debate [24,25].

Several limitations of this research should be noted. First, we did not stratify study subjects by infertility aetiology, although evidence suggests that different types of reproductive pathology might impact serum AMH in different ways. For example, ovarian response to gonadotropins is often attenuated in the setting of endometriosis, and serum AMH is reduced in fertility patients as a function of severity of the disease [26]. In contrast, polycystic ovary syndrome (PCOS) is associated with abnormally increased follicular numbers and relatively high serum AMH. This enhanced AMH production by granulosa cells in women with PCOS may be a dysfunctional manifestation of impaired access of FSH to the follicular compartment [27]. This retrospective study did not take these factors into account. Additionally, we should note that while a trend of higher median serum AMH levels was observed among patients who conceived compared to those who did not attain pregnancy, our study was not designed to detect this difference.

## Conclusions

Although others have focused on d3 serum AMH levels to predict clinical pregnancy [28], the present study aimed to assess an important intermediate IVF endpoint--blastocyst development. We confirmed a moderate positive correlation between AMH and total number of retrieved oocytes, as well as the number of MII oocytes. Our data from blastocyst culture agree with and extend previous AMH research, where AMH was noted to have a highly significant correlation with number of oocytes retrieved and fertilised where d2 and d3 embryo transfer occurs [29]. This investigation also found pre-treatment serum AMH to have moderate positive correlations with development of early cleavage-stage embryos and embryos with at least 7-cells on d3, observations generally consistent with previous reports [29,30]. The observed positive correlation between basal serum AMH and subsequent blastocyst formation will require larger sampling to refine the role of AMH in estimating embryo transfer strategies.

## Competing interests

The authors declare that they have no competing interests.

## Authors' contributions

ESS was lead investigator and organised the manuscripts; GSC assisted in study design and provided statistical analysis; ACB collected clinical and laboratory data; DJW, ACP and APHW were consultant physicians with oversight of the clinical programme; RDS was Chief-of-Service and principal project supervisor. All authors read and approved the final version.
